# The presence of the putative *Gardnerella vaginalis* sialidase A gene in vaginal specimens is associated with bacterial vaginosis biofilm

**DOI:** 10.1371/journal.pone.0172522

**Published:** 2017-02-27

**Authors:** Liselotte Hardy, Vicky Jespers, Magelien Van den Bulck, Jozefien Buyze, Lambert Mwambarangwe, Viateur Musengamana, Mario Vaneechoutte, Tania Crucitti

**Affiliations:** 1 HIV and Sexual Health Group, Department of Public Health, Institute of Tropical Medicine, Antwerp, Belgium; 2 Laboratory Bacteriology Research, Faculty of Medicine & Health Sciences, University of Ghent, Ghent, Belgium; 3 HIV/STI Reference Laboratory, Department of Clinical Sciences, Institute of Tropical Medicine, Antwerp, Belgium; 4 Clinical Trials Unit, Department of Clinical Sciences, Institute of Tropical Medicine, Antwerp, Belgium; 5 Rinda Ubuzima, Kigali, Rwanda; Massachusetts General Hospital, UNITED STATES

## Abstract

Bacterial vaginosis (BV) is a difficult-to-treat recurrent condition in which health-associated lactobacilli are outnumbered by other anaerobic bacteria, such as *Gardnerella vaginalis*. Certain genotypes of *G*. *vaginalis* can produce sialidase, while others cannot. Sialidase is known to facilitate the destruction of the protective mucus layer on the vaginal epithelium by hydrolysis of sialic acid on the glycans of mucous membranes. This process possibly facilitates adhesion of bacterial cells on the epithelium since it has been linked with the development of biofilm in other pathogenic conditions. Although it has not been demonstrated yet, it is probable that *G*. *vaginalis* benefits from this mechanism by attaching to the vaginal epithelium to initiate biofilm development. In this study, using vaginal specimens of 120 women enrolled in the Ring Plus study, we assessed the association between the putative *G*. *vaginalis* sialidase A gene by quantitative polymerase chain reaction (qPCR), the diagnosis of BV according to Nugent score, and the occurrence of a BV-associated biofilm dominated by *G*. *vaginalis* by fluorescence in situ hybridisation (FISH). We detected the putative sialidase A gene in 75% of the *G*. *vaginalis*-positive vaginal specimens and found a strong association (p<0.001) between the presence of a *G*. *vaginalis* biofilm, the diagnosis of BV according to Nugent and the detection of high loads of the *G*. *vaginalis* sialidase A gene in the vaginal specimens. These results could redefine diagnosis of BV, and in addition might guide research for new treatment.

## Introduction

*Gardnerella vaginalis* has consistently been found in bacterial vaginosis (BV) [1–3], a dysbiosis of the vaginal econiche in which the health-associated lactobacilli are outnumbered by other aerotolerant and anaerobic organisms. It has been demonstrated that a vaginal mucosa polymicrobial biofilm is associated with BV [4,5]. *G*. *vaginalis* is able to adhere to the vaginal epithelial cells and subsequently develop a biofilm on the vaginal wall [4,5], a mechanism that possibly increases the tolerance of *G*. *vaginalis* to lactic acid and hydrogen peroxide produced by lactobacilli [6] and to antimicrobial treatment [7,8]. It has been suggested that *G*. *vaginalis* initiates the colonisation of the vaginal mucosa and acts as a scaffold to which other species subsequently can attach [6,9,10]. However, *G*. *vaginalis* can also occur in the healthy vaginal microbiome (although in lower concentrations) [1,2,11], suggesting that the mere presence of *G*. *vaginalis* does not necessarily result in biofilm formation and BV. This observation has led several researchers to hypothesise that different types of *G*. *vaginalis* with different virulence potentials might exist [12–14].

Certain *G*. *vaginalis* genotypes can produce sialidase, also known as neuraminidase [13]. Sialidase is an enzyme that cleaves sialic acid from terminal glycans of glycoproteins, which are also present in the cervicovaginal fluid [15]. Sialidases have been studied in many contexts of bacterial pathogenesis, and these studies provide several examples of possible mechanisms by which these enzymes could also act in the pathogenesis of BV. In general, the production of sialidase is a common virulence factor in pathogens such as the Influenza virus [16] and a large number of bacterial species, such as *Propionibacterium acnes* [17], *Pseudomonas aeruginosa* [18], *Streptococcus pneumoniae* [19] and *Vibrio cholerae* [20]. Furthermore, sialidase has been strongly linked with bacterial biofilm development [21–23]. Cleaving off sialic acid by sialidase provides bacteria with free sialic acid that can serve as a nutrient [24], and the exposure of the underlying glycan-binding site facilitates adhesion of bacterial cells [25,26]. In addition, it has been suggested that, by incorporation of the cleaved sialic acids into bacterial cell-surface structures, bacteria could disguise themselves as host cells and bypass the host’s immune response [26,27]. Increased sialidase activity has been detected in the vaginal fluid of BV patients [28,29], and is the basis of a marketed quick test for diagnosis of BV [30]. It has been demonstrated that sialidase facilitates the destruction of the protective mucus layer in the vagina [31] and increases the proteolysis of innate immune factors such as secretory IgA [15].

Although it has not been demonstrated yet, it is probable that *G*. *vaginalis* benefits from this mechanism by attaching to the vaginal epithelium to initiate biofilm development. Although other BV-associated bacteria (e.g. *Prevotella* and *Bacteroides species*) have also been shown to produce sialidase in the vagina [28], *G*. *vaginalis* is most frequently isolated, in high concentrations, from vaginal fluid of women with BV [1–3] and has a higher tendency to adhere to vaginal epithelial cells compared to other BV-associated anaerobes [6]. We hypothesised that, like other species [21–23], the genotypes of *G*. *vaginalis* that contain the putative sialidase A gene are associated with the presence of vaginal biofilms, leading to BV. Therefore, we assessed the association between the presence of the putative sialidase A gene in *G*. *vaginalis* and the occurrence of BV-associated *G*. *vaginalis* biofilm in vaginal samples of women with and without BV.

## Methods and materials

### Study participants and ethics statement

Vaginal samples were collected from 120 Rwandan women participating in a study on the acceptability of using an intravaginal ring for contraception (NuvaRing®, Merck, New Jersey, USA) and its effect on the vaginal microbiome (the Ring Plus project [32]). Participants were between 18 and 35 years old and provided written informed consent for participation in the study. The Ring Plus project was approved by the Rwanda National Ethics Committee (Approval number 481/RNEC/2013); and the ethics committees of the Institute of Tropical Medicine (ITM), Belgium (Approval number 864/13); the Antwerp University Hospital, Belgium (Approval number 13/7/85); and the University of Liverpool, UK (Approval number RETG000639IREC).

### Vaginal sample collection

Vaginal samples were collected at the enrolment visit and at each ring removal visit by the study clinician. Two Copan flocked® swabs (Copan, Brescia, Italy) and one cotton swab were brushed against the lateral walls of the vagina. The cotton swab was immediately used to prepare two vaginal slides: one for Gram staining and one for fluorescence in situ hybridisation (FISH) on a Superfrost Plus® slide (Menzel-Gläser, Braunschweig, Germany). Both were heat-fixed by passing twice through a flame. The Superfrost Plus® slides were stored and shipped at room temperature to the ITM, where they were fixed for a second time using Carnoy solution (6:3:1, ethanol:chloroform:glacial acetic acid) [5] for 12 hours minimum. The Copan flocked® swabs were eluted by vortexing for at least 15 seconds in 1.2 ml of diluted phosphate buffered saline (PBS) (pH 7.4–1:9, PBS:saline). The eluates were stored at -80°C until shipment and shipped to the ITM using a temperature-controlled dry shipper.

### Laboratory methods

#### Nugent score

The status of the vaginal microbiome was assessed at the Rinda Ubuzima laboratory (Kigali, Rwanda) by Nugent scoring of a Gram stained vaginal slide [33]. A score of 0–3 was considered as normal vaginal microbiome; a score of 4–6 as intermediate vaginal microbiome and a score of 7–10 as BV.

#### Peptide nucleic acid fluorescence in situ hybridisation

Peptide nucleic acid (PNA) FISH on the vaginal slides using a species-specific probe for *G*. *vaginalis* (Gard162) and the broad-range BacUni-1 probe and imaging was performed as described earlier [5]. Separate scattered bacterial cells were defined as dispersed bacteria. Aggregates of bacterial cells, sticking to the vaginal epithelial cells, were defined as adherent bacteria forming a biofilm.

#### Quantitative polymerase chain reaction for *Gardnerella vaginalis*

DNA was extracted from 250 μl of the vaginal swab eluate using the Abbott m2000sp automated extraction platform (Abbott, Maidenhead, UK), according to the manufacturer’s instructions. The volume of 200 μl DNA extract was stored at –80°C until testing. qPCR was performed for each bacteria species separately, to avoid competition between the primers. The 25 μl PCR mixture contained 12.5 μl Rotor-Gene SYBR Green RT-PCR Master mix (Qiagen, Venlo, the Netherlands), 5 μl DNA extract, 1 μM of *G*. *vaginalis* forward and reverse primers targeting the 16S rRNA (Integrated DNA Technologies, Leuven, Belgium) and RNase-free water provided with the Rotor- Gene SYBR Green PCR kit. The primers for *G*. *vaginalis* and the amplification reactions (Rotor Gene Q MDx 5 plex, Qiagen) have been described before [5,11].

Quantification was done using standard curves, constructed using DNA extracts from *G*. *vaginalis* (LMG 7832^T^), grown at 35°C ± 2°C on Columbia agar base (Becton Dickinson) + 5% horse blood, under anaerobic conditions. DNA concentrations were determined using NanoDrop (Thermo Fisher Scientific, Erembodegem, Belgium) and the number of genomes was calculated using the described genome sizes and G+C content of the strains. A total of six tenfold dilutions of the DNA stocks were prepared in high performance liquid chromatography (HPLC) grade water. Both the standard curve and samples were run in duplicate. The bacterial load was expressed as genome equivalents (geq)/ml.

#### Quantitative polymerase chain reaction for the putative *Gardnerella vaginalis* sialidase A gene

The design of the primer set for amplification of the putative *G*. *vaginalis* sialidase A gene was based on previous work by Lopes dos Santos Santiago et al. [13] and on the sequence of sialidase A (NZ_ ACGF02000001.1) from the fully sequenced *G*. *vaginalis* ATCC 14019 strain (reference genome for the Human Microbiome Project, Baylor College of Medicine, Houston, TX). It should be noted that although the *G*. *vaginalis* ATCC 14019 reference strain originates from a woman diagnosed with BV and includes the putative sialidase A gene, it does not produce sialidase in laboratory cultures [31,34].

The previously designed *G*. *vaginalis* sialidase forward primer (GVSI Forward, 5’-GACGACGGCGAATGGCACGA-3’) [13] was combined with an updated reverse primer (GVSI Reverse2, 5’-TACAAGCGGCTTTACTCTTG- 3’), designed using Primer Blast (National Center for Biotechnology Information, Bethesda, MD) to eliminate the occurrence of primer dimers. The putative *G*. *vaginalis* sialidase A gene amplification was restricted to the vaginal samples containing *G*. *vaginalis* as defined by the above described qPCR. The 25 μl PCR mixture contained 12.5 μl Rotor-Gene SYBR Green qPCR Master mix (Qiagen, Venlo, the Netherlands), 5 μl DNA extract, 0.75 μM of 5 μM *G*. *vaginalis* sialidase forward and reverse primers (Integrated DNA Technologies, Leuven, Belgium) and RNase-free water provided with the Rotor-Gene SYBR Green PCR kit.

The amplification reactions were performed using the Rotor Gene Q MDx 5 plex (Qiagen, Venlo, the Netherlands) and the amplification program (10 min 95°C, (5 sec 95°C– 10 sec 58°C) x 45) was followed by melting curve analysis. Each sample was run in duplicate and each run included a standard curve. An exhaustive validation of the qPCR protocol (S1 Appendix) demonstrated that the putative *G*. *vaginalis* sialidase A gene qPCR was sensitive and specific for *G*. *vaginalis*.

### Statistical analysis

Laboratory analysis was performed blinded to all other data, except for the qPCR of the putative sialidase A gene which was performed on vaginal samples containing *G*. *vaginalis* according to qPCR.

Bacterial counts were log 10 transformed before analysis. Data analysis was done using STATA13. The p-values reported for associations between the presence and quantity of the sialidase gene and BV/qPCR-biofilm results were obtained using mixed effects ordered logistic regression.

## Results

### Characterisation of vaginal samples

**Fig 1:**
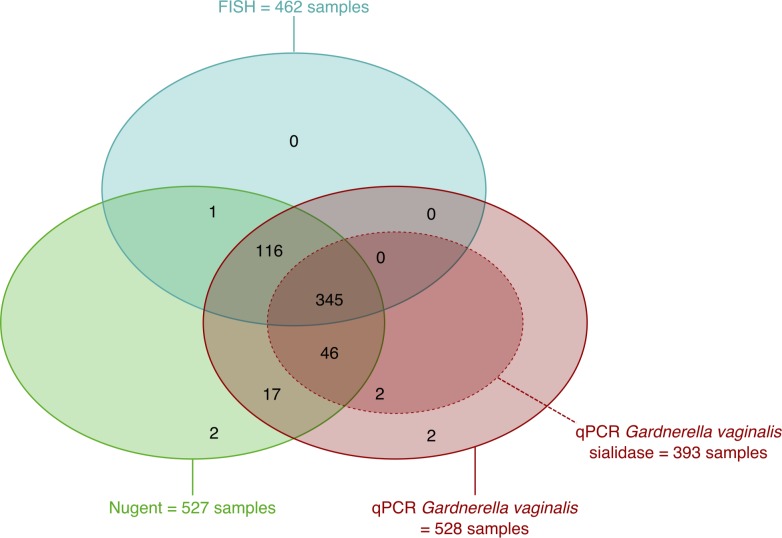
Overview of different subsets of samples analysed with fluorescence in situ hybridisation (n = 462), Nugent score (n = 527), *G*. *vaginalis* quantitative polymerase chain reaction (n = 528) and *G*. *vaginalis* sialidase quantitative polymerase chain reaction (n = 393).

A total of 527 samples were available for Nugent scoring, 462 samples were analysed by FISH and 528 samples were used for qPCR to detect *G*. *vaginalis*. All 393 *G*. *vaginalis* qPCR-positive samples were tested for the presence of the putative *G*. *vaginalis* sialidase A gene by qPCR (Fig 1, Table 1).

**Table 1:** Characteristics of vaginal samples.

Test	Total	Result	N(%)
**Nugent score**	527	0–3	299 (56.7)
		4–6	53 (10.1)
		7–10	175 (33.2)
**Fluorescence in situ hybridisation**	462	*Gardnerella vaginalis* positive	290 (62.8)
		*G*. *vaginalis* biofilm	191 (41.3)
		*G*. *vaginalis* dispersed only	99 (21.4)
**Quantitative polymerase chain reaction**	528	*G*. *vaginalis* positive	393 (74.4)
	393	*G*. *vaginalis* sialidase positive	294 (74.8)

#### Nugent score

Of the total of 527 samples of 120 participants, 299 (56.7%) showed a healthy microbiome (Nugent score 0–3), 53 (10.1%) were categorised as intermediate (Nugent score 4–6) and 175 (33.2%) were diagnosed as BV (Nugent score 7–10).

#### Fluorescence in situ hybridisation

**Fig 2:**
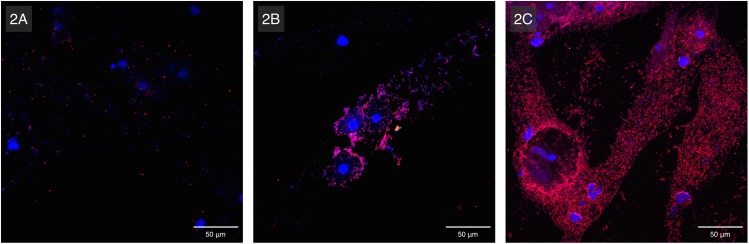
Superimposed confocal laser scanning microscopy images with 400x magnification of *Gardnerella vaginalis* biofilm, in three vaginal samples: vaginal epithelial cells DAPI in blue and *G*. *vaginalis* specific PNA-probe Gard162 with Alexa Fluor 647 in red. 2A shows an example of dispersed-only *G*. *vaginalis* (negative for biofilm), 2B shows a light *G*. *vaginalis* biofilm (a small number of bacteria are adhering to the vaginal epithelial cells) and 2C is an example of a heavy *G*. *vaginalis* biofilm (the vaginal epithelial cells are covered by bacteria).

A subset of 462 samples was analysed with FISH. The remaining 65 samples could not be analysed mainly due to the absence of epithelial cells and bacteria on the slides. *G*. *vaginalis* was present in 290 samples (62.8%) using FISH. In 191 of 290 vaginal slides (65.9%), aggregated bacteria were attached to the vaginal epithelium and considered to be part of a biofilm, although dispersed bacteria were present as well (Fig 2). In the remaining 99 vaginal slides (34.1%), *G*. *vaginalis* was only present in the planktonic/dispersed form.

#### Quantitative polymerase chain reaction

A total of 528 samples were available for molecular quantification of *G*. *vaginalis* genome equivalents. *G*. *vaginalis* was detected in 393 samples (74.4%), with a mean bacterial load (log 10) of 6.97 ± 1.37 (standard deviation) geq/ml. Moreover, the presence of the putative *G*. *vaginalis* sialidase A gene was assessed in all 393 *G*. *vaginalis*-qPCR positive samples and was detected in 294 samples (74.8%). The putative *G*. *vaginalis* sialidase A gene concentration was <106 geq/ml (low load) in 112 samples (28.5%) and ≥ 106 geq/ml (high load) in the remaining 182 samples (46.3%).

### Association between the presence of *G*. *vaginalis*, BV and biofilm according to FISH

The presence of *G*. *vaginalis* in the sample, as assessed by qPCR, was associated with the diagnosis of BV and presence of *G*. *vaginalis* biofilm, as assessed with FISH (p<0.001) (Table 2). A higher concentration of *G*. *vaginalis* in the sample was associated with the diagnosis of BV and the presence of *G*. *vaginalis* biofilm.

**Table 2:** The association between quantitative polymerase chain reaction results for *G*. *vaginalis* and *G*. *vaginalis* sialidase of vaginal samples and fluorescence in situ hybridisation and Nugent score results of vaginal slides.

	*Gardnerella vaginalis* 0 geq/ml^1^ N (%)	*G*. *vaginalis* >0 and <106(geq/ml)N (%)	*G*. *vaginalis*≥106 geq/ml N (%)	P-value^3^	*G*. *vaginalis* sialidase 0 geq/ml N (%)	*G*. *vaginalis* sialidase >0 and <106(geq/ml N (%)	*G*. *vaginalis* sialidase ≥106 geq/ml N (%)	P-value
**FISH**^2^ ***G*. *vaginalis***	**116 (100)**	**97 (100)**	**248 (100)**	**<0.001**	**86 (100)**	**96 (100)**	**163 (100)**	**<0.001**
***G*. *vaginalis* absent**	75 (64.7)	58 (59.8)	38 (15.3)		47 (54.7)	33 (34.4)	16 (9.8)	
***G*. *vaginalis* dispersed only**	23 (19.8)	23 (23.7)	53 (21.4)		24 (27.9)	28 (29.2)	24 (14.7)	
***G*. *vaginalis* biofilm**	18 (15.5)	16 (16.5)	157 (63.3)		15 (17.4)	35 (36.5)	123 (75.5)	
**Nugent score**	**135 (100)**	**109 (100)**	**282 (100)**	**<0.001**	**98 (100)**	**112 (100)**	**181 (100)**	**<0.001**
**Nugent 0–3**	120 (88.9)	98 (89.9)	80 (28.4)		80 (81.6)	61 (54.5)	37 (20.4)	
**Nugent 4–6**	3 (2.2)	5 (4.6)	45 (15.9)		8 (8.2)	14 (12.5)	28 (15.5)	
**Nugent 7–10**	12 (8.9)	6 (5.5)	157 (55.7)		10 (10.2)	37 (33.0)	116 (64.1)	

^1^ geq/ml = genome equivalent/ml; results from quantitative polymerase chain reaction.

^2^ FISH: Fluorescence in situ hybridisation.

^3^ P-values obtained by mixed effects ordered logistic regression.

### Association between the presence of the putative *G*. *vaginalis* sialidase A gene and biofilm

Both FISH and *G*. *vaginalis* sialidase A qPCR analysis were carried out for a subset of 345 samples containing *G*. *vaginalis* as assessed by qPCR. Based on our data, the presence of the *G*. *vaginalis* sialidase A gene, as assessed by qPCR, was associated with the presence of *G*. *vaginalis* biofilm, as assessed with FISH (p<0.001) (Table 2). For the 163 samples with a high load of *G*. *vaginalis* harbouring the sialidase A gene (i.e. ≥106 geq/ml), *G*. *vaginalis* biofilm was present in 75.5%, whereas dispersed-only *G*. *vaginalis* was present in 14.7% of the samples. Using FISH, *G*. *vaginalis* was not visualised in 9.8% of these samples. In the 96 samples with a low load of *G*. *vaginalis* harbouring the sialidase A gene (<106 geq/ml), a more equal distribution between the three categories was observed; with 36.5% samples with visible biofilm, 29.2% samples with only dispersed *G*. *vaginalis* and 34.4% samples with no *G*. *vaginalis* observed by FISH. Out of the 86 samples without putative *G*. *vaginalis* sialidase A gene as assessed by qPCR, *G*. *vaginalis* biofilm was detected using FISH in 17.4% of the samples, while in 27.9% samples only dispersed/planktonic *G*. *vaginalis* were seen. *G*. *vaginalis* was not observed in 54.7% of the samples.

### Association between the presence of the putative *G*. *vaginalis* sialidase A gene and the diagnosis of bacterial vaginosis

A subset of 391 samples was analysed by both Nugent scoring and putative *G*. *vaginalis* sialidase A gene qPCR. The probability of having BV according to Nugent is increased when the putative *G*. *vaginalis* sialidase A gene is present in high loads (≥ 106 geq/ml) (p<0.001) (Table 2). Of the 181 samples with a high load of the putative *G*. *vaginalis* sialidase A gene, 64.1% were BV-positive (Nugent score 7–10), 20.4% had a healthy vaginal microbiome (Nugent score of 0–3) and 15.5% were diagnosed with an intermediate Nugent score of 4–6. In contrast, BV was diagnosed in 10 out of the 98 samples (10.2%) in which no putative *G*. *vaginalis* sialidase A gene could be detected. However, 80 of 98 (81.6%) samples represented a healthy vaginal microbiome according to Nugent and 8 of 98 samples (8.2%) had an intermediate score. Additionally, when the putative *G*. *vaginalis* sialidase A gene was present in low amounts (<106 geq/ml), 54.5% of the 112 samples were considered healthy according to the Nugent score, 33.0% were categorised as BV, and 12.5% represented an intermediate vaginal microbiome.

## Discussion

BV is the most prevalent vaginal disorder in women of reproductive age worldwide, and aside from the discomfort in case of symptomatic BV, it can also generate an array of serious gynaecological and obstetric complications. The presence of BV-associated anaerobes in the vaginal environment increases the risk for preterm labour and birth [35]. Furthermore, the presence of sialidase in vaginal fluid has been linked to BV and to preterm birth as well [36,37]. In a large cohort of 1806 women that included 800 women with BV and 53 spontaneous preterm births, Cauci et al. [37] showed that the sialidase levels in the vaginal fluid were significantly associated with all adverse pregnancy outcomes.

*G*. *vaginalis* plays an important role in BV, since *G*. *vaginalis* overgrowth is found in nearly all cases of BV [38]. However, the presence of *G*. *vaginalis* in healthy vaginal environments [11,39] contradicts its pathogenic role in BV. To resolve this discrepancy, it has been suggested that *G*. *vaginalis* might actually consist of different subspecies with distinct roles in BV pathogenesis, which is supported by the genotypic and phenotypic diversity of the species [14,34,40]. Although other BV-associated bacteria (e.g. *Prevotella* and *Bacteroides* species) are able to produce sialidase [28], we decided to investigate *G*. *vaginalis* sialidase in BV, considering that *G*. *vaginalis* is most frequently isolated from vaginal fluids of women suffering from BV [1–3,38] and that it has a higher tendency to adhere to vaginal epithelial cells *in vitro* compared to other BV-associated anaerobes [6]. We studied the association between the presence of the putative *G*. *vaginalis* sialidase A gene, as a proxy for sialidase production, in the vagina and the occurrence of BV and bacterial biofilm on the vaginal epithelium. To this end, we screened vaginal samples of 120 Rwandan women [32] by means of a putative *G*. *vaginalis* sialidase A specific qPCR and assessed the occurrence of BV by means of Nugent scoring and of biofilm by confocal laser scanning microscopy (CLSM) after FISH for *G*. *vaginalis* on vaginal samples.

In this population, the putative *G*. *vaginalis* sialidase A gene was detected in about 75% of the *G*. *vaginalis*-positive samples. In about 60% of those samples, a high load (≥10^6^ geq/ml) of the gene was detected. This high prevalence of the *G*. *vaginalis* sialidase A gene in our study may be explained by the cohort of women enrolled for this study and the high prevalence of vaginal dysbiosis, i.e. in 43.3% of all samples. Earlier studies have investigated the presence of the *G*. *vaginalis* sialidase gene and the production of sialidase in cultured isolates [13,28,41,42]. Using clinical isolates from Belgian women, Lopes dos Santos Santiago and colleagues [13] detected the *G*. *vaginalis* sialidase A gene using qPCR in 51% of the strains. When using the filter paper spot test for the detection of sialidase activity, von Nicolai et al. [41] could detect sialidase production in only 1 of 10 clinical isolates. Additionally, Briselden et al. [28] detected sialidase activity in 20% of 105 *G*. *vaginalis* isolates with no difference in isolates from women with and without BV), and Moncla and Pryke [42] observed sialidase activity in 39% of 31 isolates.

At present, it is not clear whether the sialidase gene is expressed constitutively or not. Pleckaityte and colleagues [43] detected a sialidase gene in 17 tested *G*. *vaginalis* isolates, but only 10 of these strains actually produced sialidase *in vitro*. Schellenberg et al. [34] also found that the gene presence was not predictive of actual sialidase activity using a filter spot assay: out of 77 *G*. *vaginalis* isolates positive for the sialidase gene, 36 produced sialidase. In addition, almost all these sialidase-producers [33] belonged to the same chaperonin-60 universal target-based molecular subgroup, a group that consisted solely of sialidase-producers [34]. In currently ongoing (not yet published) *in vitro* experiments by our group, we found that only 29 out of 41 sialidase A gene- positive *G*. *vaginalis* isolates produced sialidase, based on the filter spot test. Interestingly, we noticed that all but two sialidase-producing strains were isolated from women with BV according to Nugent. This contradicts what was published by Lopes dos Santos Santiago et al. [13] who found a 100% correspondence between the mere presence of the gene and sialidase activity in 19 *G*. *vaginalis* isolates. However, all but one of these isolates were obtained from women with a disturbed microbiome, which might have introduced a bias [13]. The absence of sialidase activity in *G*. *vaginalis* isolates containing the sialidase gene might be explained by the absence of an alternative gene encoding this activity or the need for other factors to stimulate the expression of the gene. The presence of sialic acid on epithelial cells might be a possible trigger that activates the sialidase gene and subsequent production of sialidase. It could also be possible that a threshold in *G*. *vaginalis* concentration needs to be reached in order to activate the sialidase gene. In any case, more basic research is needed to fully understand the sialidase expression pathway.

When looking at the association between the presence of the *G*. *vaginalis* and its sialidase gene and the diagnosis of BV by Nugent score, we found that the probability for having BV (Nugent score 7–10) was increased when a high concentration of *G*. *vaginalis* and its putative sialidase A gene was present in the vaginal samples. This was expected, since sialidase production by *G*. *vaginalis* is recognised as a virulence factor [43], and has already been associated with BV [37]. In our previous work [5], we confirmed the importance of *G*. *vaginalis* in the development of a biofilm on the vaginal epithelium in BV, as established by Swidsinski et al. [4] in 2005.

Ours was the first study to use clinical samples to demonstrate the significance of *G*. *vaginalis*’ potential ability to produce sialidase and to document its association with BV and vaginal biofilm. We established a strong association between a high load of the putative *G*. *vaginalis* sialidase A gene, as measured by a specific qPCR, and *G*. *vaginalis* being part of a vaginal epithelium biofilm, visualised by CLSM after FISH. Sialidase has been linked with biofilm development in other microorganisms. In *Pseudomonas aeruginosa*, sialidase (or neuraminidase) contributes to the initial colonisation of the airway, and colonisation could be blocked *in vitro* by viral neuraminidase inhibitors [21]. Likewise in pneumococcal infections, sialidase is involved in biofilm formation and pathogenesis of respiratory tract infections [22,23]. Also, sialidase producing *Propionibacterium acnes* isolates were more associated with acne than sialidase negative isolates [44].

A shortcoming of this study is the absence of isolates. Clinical isolates would have provided valuable information on ARDRA genotyping and the actual sialidase activity. Being able to assess sialidase production by *G*. *vaginalis* directly in our Ring Plus samples would have been interesting, but since sialidase activity in the vaginal samples could also have resulted from other vaginal bacterial species, it would have confounded the results. Despite this limitation, we were able to establish that the potential ability of *G*. *vaginalis* to produce sialidase is linked to the presence of BV and the existence of a vaginal *G*. *vaginalis* biofilm. This finding may improve BV diagnosis, but it may also guide future research for new and better treatments for this recurrent and difficult-to-treat condition. Future studies should investigate biofilm-formation linked to sialidase-production in different subtypes of *G*. *vaginalis*.

## Supporting information

S1 Appendix Validation Gv sialidase results. (XLSX)
